# Emerging Roles of Lipophagy in Health and Disease

**DOI:** 10.3389/fcell.2019.00185

**Published:** 2019-09-10

**Authors:** Konstantinos Kounakis, Manos Chaniotakis, Maria Markaki, Nektarios Tavernarakis

**Affiliations:** ^1^Institute of Molecular Biology and Biotechnology, Foundation for Research and Technology – Hellas, Heraklion, Greece; ^2^Department of Basic Sciences, Medical School, University of Crete, Heraklion, Greece; ^3^Department of Chemistry, University of Crete, Heraklion, Greece

**Keywords:** lipophagy, lipid droplet, fatty acids, aging, fatty liver disease, fibrosis, cancer, obesity

## Abstract

The term lipophagy is used to describe the autophagic degradation of lipid droplets, the main lipid storage organelles of eukaryotic cells. Ever since its discovery in 2009, lipophagy has emerged as a significant component of lipid metabolism with important implications for organismal health. This review aims to provide a brief summary of our current knowledge on the mechanisms that are responsible for regulating lipophagy and the impact the process has under physiological and pathological conditions.

## Introduction

### Autophagy at a Glance

The term autophagy is used to describe the lysosomal degradation of cytosolic material, a highly conserved process. It encompasses three distinct but related types: macroautophagy, microautophagy and chaperone-mediated autophagy (CMA). Macroautophagy involves the sequestration of cytoplasmic components (including organelles) into a double-membrane structure known as autophagosome, which eventually fuses with late endosomes and lysosomes for subsequent breakdown of its cargo. Microautophagy involves the direct engulfment of cellular components by invaginations of the lysosomes. Finally, CMA involves the recognition and capture of cytosolic components by Hsc70 chaperones and the subsequent translocation of these components into the lysosomal lumen (Mizushima et al., 2011; Rogov et al., 2014).

Autophagy can act as a means for the cell to redistribute valuable nutrients in conditions of starvation; this is the case of bulk autophagy, which generally targets random parts of the cytosol. It can also act as a means to dispose of excessive or damaged organelles or invading microbes; this is the case of selective autophagy that involves the degradation of specific organelles such as the nucleus, mitochondria, peroxisomes, and often relies on specialized receptors and regulatory pathways to achieve that specificity (Rogov et al., 2014).

### Lipophagy as a Subset of Autophagy

Lipid droplets (LDs) are eukaryotic organelles responsible for the storage of lipids in the forms of triacylglycerols (TAGs), cholesteryl esters and retinyl esters surrounded by a phospholipid monolayer. The surface of the droplets is also coated with proteins, such as the perilipins; these are a family of five proteins that contribute to lipid droplet packing and regulate interactions with other organelles, droplet size, and accessibility to lipolytic mechanisms. Other LD proteins include enzymes involved in triacylglycerol and phospholipid synthesis or lipid transporters. The size of LDs can be quite varied, ranging from 1 μm in the majority of cell types to 100 μm in white adipocytes (Moore et al., 2005; Marcinkiewicz et al., 2006; Reue, 2011; Kimmel and Sztalryd, 2016).

Lipid droplets allow for the storage of lipids that can be utilized for energy production as well as the synthesis of membrane components, signaling ligands and other special macromolecules. Such lipid storage protects the cells from exposure to excessive amounts of free fatty acids (FFAs) and sterols that can be damaging to cellular membrane composition, signaling pathways and metabolic homeostasis (Reue, 2011; Kimmel and Sztalryd, 2016). The lipids can be accessed when needed through the process of lipolysis, which involves the breakdown of TAGs and esters by cytosolic lipases, such as Adipose Triglyceride Lipase (ATGL) (Zimmermann et al., 2004; Ong et al., 2011; Obrowsky et al., 2013).

In addition to lipolysis, lipid stores can also be accessed via lipophagy, a specific subset of selective autophagy that targets LDs and catabolizes their components into FFAs and glycerol. It was discovered in 2009 in a study that demonstrated clear co-localization of autophagic and LD markers, in conjunction with a necessity of autophagy for LD and triglyceride (TG) clearance in hepatocytes, both *in vitro* and *in vivo* (Singh et al., 2009).

In this review, we provide a brief summary of the existing knowledge on the mechanisms and regulation of lipophagy, as well as its functional importance in normal aging and disease.

### Mechanisms and Regulation of Lipophagy

Lipophagy, as any form of selective autophagy, primarily begins with the recognition of the cargo by the autophagosomal membrane through interaction with microtubule- associated protein 1 light chain 3 (MAP1LC3), which is a mammalian homolog of yeast Atg8 and a core component of the phagophore) (Maus et al., 2017). This typically involves the assistance of one or more cargo adaptors such as p62, Optineurin, NBR1 and NDP52 that connect the organelle membrane with LC3, and may require polyubiquitination of proteins on the organelle surface as a recruiting signal (Rogov et al., 2014). The exact proteins facilitating these steps of LD recognition are not entirely known, but some clues exist: Huntingtin has been shown to be necessary for lipophagy under stress conditions, and seems to act by connecting p62 with LC3-II as well as releasing the pro-autophagic kinase ULK-1 from mTOR inhibition (Rui et al., 2015). Ancient Ubiquitous Protein 1 (AUP1) is a factor that localizes to LDs and acts as a recruiter for the E2 ubiquitin conjugase G2 (Spandl et al., 2011). Proteins of the Rab molecular switch family may also play a part in this process, as many of them have been detected on LDs (Kiss and Nilsson, 2014) and some have been associated with autophagy regulation; in particular, Rab7 (Schroeder et al., 2015), Rab10 (Li et al., 2016), and Rab25 (Zhang et al., 2017) have been shown to be indispensable for lipophagy in hepatocytes under certain conditions. The cytosolic lipolysis associated lipase ATGL, (also known as PNPLA2) has been shown to be a necessary and sufficient positive regulator of lipophagy induction in mice livers (acting through the deacetylase SIRT1), suggesting tight co-ordination between two lipolytic pathways. ATGL cannot facilitate LD catabolism without lipophagy and possesses LIR motifs (LC3-II interaction motifs) that are needed for its recruitment on LDs and the initiation of lipolysis (Martinez-Lopez et al., 2016; Sathyanarayan et al., 2017). Another lipase of the same family, PNPLA8, can also interact with LC3 and induce lipophagy as part of a SREBP-2 response in a high fat diet mouse model (Kim et al., 2016). PNPLA3 is needed for lipophagy in starved human hepatocytes (Negoita et al., 2019). PNPLA5 has also been shown to contribute to the autophagy of LDs as well as autophagic proteolysis and mitophagy (Dupont et al., 2014). In addition to their potential role in LD recognition, all these lipases might contribute to lipophagy initiation by directly contributing to autophagosome formation via the mobilization of triglycerides and steryl esters (Shpilka et al., 2015; Ward et al., 2016).

Depending on their size, LDs can be targeted either by macroautophagy, in which an entire small LD is trapped in an autophagosome and consumed as a whole, or by the so-called piecemeal microautophagy, in which the autophagosomes sequester only parts of a large droplet, which then pinches off as a double-membrane vesicle enriched in LC3 for gradual consumption (Singh et al., 2009). Either way, upon lysosomal engulfment, the contents of the LD are broken down by special lipases known as lysosomal acid lipases (LALs) that are capable of catabolizing triacylglycerides, diacylglycerides, cholesteryl esters and retinyl esters (Warner et al., 1981; Sheriff et al., 1995; Grumet et al., 2016; Schulze et al., 2017b). These lipases are notably different from their cytosolic counterparts because of their ability to function in an acidic (pH = 4, 5-5) rather than neutral (pH = ∼7) environment (Zechner et al., 2017). It is worth noting that since, as mentioned previously, LDs are coated with perilipins, these proteins need to be removed before LD degradation by autophagy or even cytosolic lipases can occur. There is evidence indicating that this happens through chaperone-mediated autophagy of the perilipins themselves (Kaushik and Cuervo, 2015), a process regulated by AMPK signaling (Kaushik and Cuervo, 2016).

**FIGURE 1:**
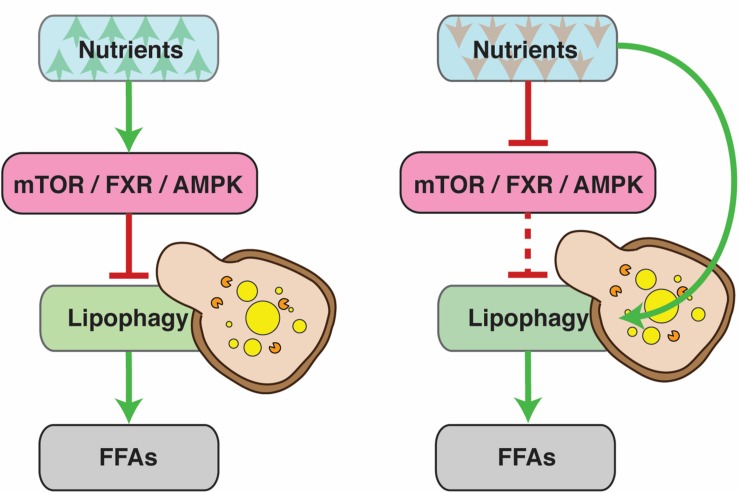
Under normal conditions, lipophagy is regulated by the nutrient status of the cell. Nutrient abundance inhibits lipophagy, since cells have no need for free fatty acids (FFAs) as an energy source. During periods of nutrient deprivation, on the other hand, lipophagy is activated to allow cells to tap into their fat reserves. The dashed line denotes reduced pathway functionality due to upstream inhibition.

At the highest level, lipophagy (and general autophagy) is regulated by systems that sense and respond to the nutrient status of the cell, such as the nuclear receptors farnesoid X receptor (FXR) and peroxisome proliferator-activated receptor alpha (PPARα), the transcriptional activator cAMP response element-binding protein (CREB) (Lee et al., 2014; Seok et al., 2014), mTOR (Lapierre et al., 2011; Lin et al., 2013; Zhang H. et al., 2018) and AMPK (Seo et al., 2017; Li et al., 2019). These systems control downstream factors in order to ensure that the levels of FFAs in the cells match their energy requirements. Generally, nutrient abundance inhibits lipophagy, while deprivation promotes it (Figure 1). Conditions that demand higher energy expenditure, such as the deregulation of RNA polymerase III, can also have an inducing effect (Bonhoure et al., 2015; Willis et al., 2018). Lipophagy can also act as a defensive mechanism against lipotoxicity. For instance, it is upregulated in models of SOCE (Store-Operated Calcium Entry) deficiency that exhibit reduced levels of cytosolic lipolysis. In this case, lipophagy is indispensible for cell survival (Maus et al., 2017).

Expression of lipophagy-associated genes is primarily controlled by the master regulator of lysosomal biogenesis transcription factor EB (TFEB)/helix-loop-helix (HLH) -30; this factor has been shown to directly induce lysosomal lipase expression under starvation conditions in *Caenorhabditis elegans* and mice hepatocytes (O’Rourke and Ruvkun, 2013) and it is required for LD clearance in multiple systems (O’Rourke and Ruvkun, 2013; Settembre et al., 2013). Other factors that have been linked to lipophagy regulation are TFE3, which can induce lipophagy in hepatocytes (Xiong et al., 2016) and the forkhead homeobox transcription factor FOXO1, which is involved in lysosomal lipase and lipophagy induction in murine adipocytes under nutrient restricted conditions (Lettieri Barbato et al., 2013). FOXO1 is also needed for lipophagy in hepatocytes, in conjunction with FOXO3 and FOXO4 (Xiong et al., 2012).

## The Significance of Lipophagy

### Lipophagy and Aging

Experiments in *C. elegans* have shown that HLH-30/TFEB- mediated autophagy is a critical component of long lived phenotypes (Lapierre et al., 2013) and that the induction of lysosomal lipases can have a positive effect on organismal lifespan (O’Rourke and Ruvkun, 2013). LIPL-4, in particular, is a lipase that is necessary for longevity in germline-less worms, and whose overexpression extends lifespan in an autophagy dependent fashion (Lapierre et al., 2011). It seems that it does not actively participate in lipophagy, but may induce it by causing an enrichment of ω-3/6 polyunsaturated fatty acids (O’Rourke et al., 2013). Downstream of LIPL-4 lies a signaling cascade in which a free fatty acid, oleoylethanolamide (OEA), activates nuclear receptors NHR-49 and NHR-80 to promote longevity (Folick et al., 2015).

### Lipophagy and Disease

#### Alcoholic Fatty Liver Disease

**FIGURE 2:**
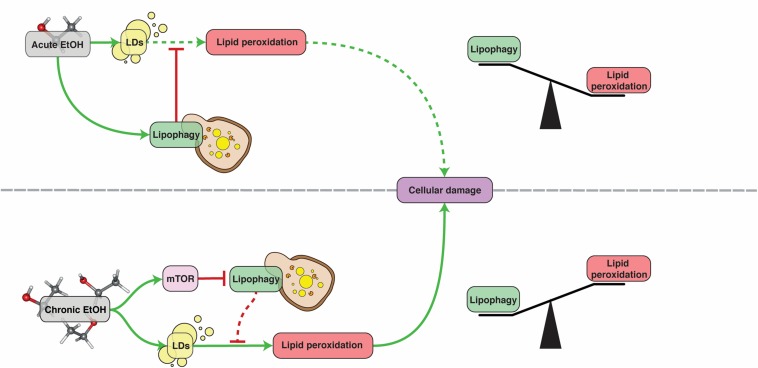
The role of lipophagy in AFLD. Under acute ethanol (EtOH) exposure, lipophagy acts as a defense mechanism against lipid peroxidation, protecting hepatocytes. Under chronic exposure however, lipophagy is inhibited by mTOR and can no longer provide this protection. See text for more details. Dashed lines denote reduced pathway functionality due to upstream inhibition.

Alcoholic fatty liver disease (AFLD) refers to the damage caused to the liver by excessive consumption of alcohol. The symptoms include oxidative stress, lipid droplet accumulation in the cytoplasm of hepatocytes, mitochondrial damage and cell death (Ding et al., 2011b). In particular, steatosis (excessive LD deposition) is an extremely common symptom of alcohol abuse (O’Shea et al., 2010). There are very few options for treatment of this disease and, despite the excessive research done to understand its pathophysiology, there is no therapy available and the treatment remains almost the same as 50 years ago (Singh et al., 2017). AFLD is a multifactorial disease that involves interactions between lipid metabolism, the immune system and oxidative stress (Livero and Acco, 2016). Alcohol oxidation in the liver induces lipid accumulation by shifting the cells redox potential from fatty acid β-oxidation to reductive synthesis. It also transcriptionally induces lipogenic enzyme expression and promotes import from fatty acid transporters (You and Arteel, 2019). It has been found that, upon short-term supplementation, ethanol induces mitophagy and lipophagy in hepatocytes, which likely act as repair mechanisms against damage caused to mitochondria and steatosis (Ding et al., 2011a). Ethanol-mediated mitochondrial damage leads to an increase in intracellular ROS, which can induce autophagy through Beclin-1 as a means to combat oxidative damage (Tang et al., 2016). Nonetheless, chronic alcohol exposure causes an impairment of autophagy and lipophagy (Donohue et al., 1994, Donohue et al., 2007; Ding et al., 2011a, b; Schulze et al., 2017a; Chao et al., 2018). This impairment is most likely caused by activation of mTOR signaling and a reduction of lysosomal biogenesis in mice hepatocytes. Administration of the mTOR inhibitor Torin-1 to these mice restores lysosomal biogenesis and decreases steatosis and liver damage (Chao et al., 2018). Induction of autophagy with resveratrol in mice or HepG2 cell lines was similarly beneficial (Tang et al., 2016). In conclusion, lipophagy acts as a defensive mechanism against AFLD but cannot exert its protective activity for long as it is inhibited by chronic alcohol consumption (Figure 2).

#### Non-alcoholic Fatty Liver Disease

Non-alcoholic fatty liver disease (NAFLD) is an umbrella term used for numerous liver- related conditions characterized by the accumulation of triglycerides in hepatocytes that is caused by an upregulation of free fatty acid levels and lipogenesis (Alkhouri et al., 2009). NAFLD generally involves insulin resistance and the redirection of glucose from glycogen synthesis to lipogenesis (Samuel et al., 2004; Irimia et al., 2017). It has been associated with mutations in various lipid metabolism genes, such as patatin-like phospholipase domain-containing-3 (PNPLA3) (Romeo et al., 2008), transmembrane 6 superfamily member 2 (TM6SF2) (Mahdessian et al., 2014), membrane bound O-acyltransferase domain containing 7 (MBOAT7) (Luukkonen et al., 2016) and multiple apolipoprotein C3 (ApoC3) gene variants (Petersen et al., 2010; Peter et al., 2012; Zhang R.-N. et al., 2016). It has also been associated with mutations in autophagy-related genes. One such case is a deletion in the immunity-related GTPase family M (IRGM) gene that inhibits autophagic flux and increases LD availability in HepG2 and PLC/PRF/5 cells. This phenotype can be rescued via treatment with the autophagy inducer rapamycin (Lin et al., 2016). Superoxide dismutase 1 (SOD1) knock out mice exhibit low visceral fat and an increase of liver LDs during fasting that results in liver damage. Lipophagy is impaired in this model, with p62 accumulating on LDs but failing to complete the process (Kurahashi et al., 2015). Sterol regulatory element-binding protein 2 (SREBP-2) is observed to be activated unconventionally to promote excessive cholesterol accumulation in NAFLD. Inhibition of SREBP-2 activity in cell and mouse models of NAFLD upregulated expression of autophagic markers, ultimately reducing lipid deposition (Deng et al., 2017). Induction of autophagy by FGF21 supplementation was successful in treating the NAFLD phenotype in fat-loaded HepG2 cells (Zhu et al., 2016). Knock down of Rubicon, an autophagy suppressor that is increased in NAFLD patients, via siRNA in HepG2 cells, BNL-CL2 cells, and murine primary hepatocytes attenuated autophagy impairment and reduced endoplasmic reticulum stress, apoptosis and lipid accumulation in NAFLD inducing conditions. These positive effects were replicated in Rubicon KO mice (Tanaka et al., 2016). Recently, it was shown that serum methionine levels were abnormally increased in human NAFLD patients. Moreover, autophagy and lipophagy were impaired in hepatocytes from glycine *N*-methyltransferase (GNMT) -KO mice. Interestingly, a methionine deficient diet could rescue liver steatosis and restore autophagy levels to normal in GNMT-KO mice (Zubiete-Franco et al., 2016). All in all, the majority of studies indicate that lipophagy counteracts the progression of NAFLD, but is abnormally inhibited in some instances of the disease. However, it is worth noting that there is one study suggesting otherwise, as it showed that suppression of autophagy through inhibition of c-Jun N-terminal Kinase (JNK) attenuates insulin resistance in a NAFLD rat model (Yan et al., 2017).

#### Liver Fibrosis

The term liver fibrosis is used to describe the common pathways of chronic or iterative damage that can be afflicted onto the liver by toxic factors, viral infections, autoimmune conditions, or metabolic and aging aspects (Campana and Iredale, 2017). Progression of the disease leads to the emergence of liver fiber nodules and the disruption of liver structure and function by excessive deposition of extracellular matrix (ECM) components (Zhang Z. et al., 2018). Hepatic stellate cell (HSC) activation plays a pivotal role in this process by secreting fibrogenic factors that promote the production of collagen and the propagation of fibrosis (Zhang C.-Y. et al., 2016). Contrary to the previously mentioned diseases, evidence shows that lipophagy fuels fibrosis in the liver and potentially other tissues. Characteristic marks of HSC activation include the release of extracellular lipid droplet contents, endoplasmic reticulum stress, oxidative stress, overexpression of G proteins and accumulation of p62. Interestingly, lipid droplet accumulation or inhibition of mitochondrial fatty acid β-oxidation inhibits fibrosis (Hernandez-Gea et al., 2012, 2013; Kim et al., 2018). Blockage of autophagy through RNA interference or bafilomycin A1 reduced fibrogenesis and ECM accumulation in mouse and human HSC lines (Thoen et al., 2011; Hernandez-Gea et al., 2012). Lipophagy in HSCs has been shown to be partially mediated through Rab25in a ROS dependent manner. Antioxidants, such as glutathione and N-acetyl cysteine, were effective in halting autophagy, suggesting a potential therapeutic approach (Zhang et al., 2017).

#### Lipophagy and Cancer

Tumors grow in a unique microenvironment with insufficient supply of oxygen and nutrients. Survival in such an environment requires metabolic reprogramming. A significant part of this reprogramming involves changes in lipid metabolism, with aggressive tumors exhibiting increased acquisition, production and storage of lipids and lipoproteins. Fatty acid β-oxidation provides a significant source of energy for tumors and is the dominant bioenergetic pathway for non-glycolytic tumors (Beloribi-Djefaflia et al., 2016). Autophagy has been shown to have both pro-and anti-cancer roles. For instance, it can be anti-oncogenic by inhibiting inflammation or pro-oncogenic by protecting tumor cells from ROS damage due to hypoxic stress and preventing necrotic cell death (Degenhardt et al., 2006; Yazdani et al., 2019).

Similarly, lipophagy can also have a dual role in cancer growth. On the one hand, lipophagy contributes to the mobilization of stored lipid content, allowing tumor cells to access a supply of energy that can be critical to their growth (Gomez de Cedron and Ramirez de Molina, 2016). CCAAT enhancer binding protein α (C/EBPα), a protein that is upregulated in hepatocellular carcinoma (HCC) patients, promotes resistance to energy starvation and carcinogenesis through lipophagy (Lu et al., 2015). On the other hand, lipophagy has been shown to act against tumorigenesis. Lysosomal acid lipase (LAL), the lipase that facilitates lipophagy, has been found to exhibit tumor suppressor activity, as its deficiency permits cancer growth and metastasis through the mTOR dependent activation of myeloid-derived suppressor cells (Zhao et al., 2015). Hepatocyte specific expression of human LAL in otherwise LAL deficient mice was sufficient to inhibit B16 melanoma metastasis in the liver and lung (Du et al., 2015). Lipophagy has also been shown to cause apoptosis in HeLa cells through the induction of endoplasmic reticulum and mitochondrial stress (Mukhopadhyay et al., 2017). Rab7, already mentioned as a lipophagy regulator, has been shown to have potential tumor suppressive properties and inhibit prostate tumor growth and invasion (Steffan et al., 2014).

#### Lipophagy in Obesity

A concrete link between lipophagy and obesity has still to be identified, although there are many indicators for such a relationship. Autophagy is generally downregulated in high fat diet models (Koga et al., 2010; Rodriguez-Navarro et al., 2012). Defective hepatic autophagy in obese mice induces insulin resistance through the promotion of endoplasmic reticulum stress. Restitution of autophagy through overexpression of Atg7 in these mice can restore insulin levels back to normal and improved glucose tolerance (Yang et al., 2010). In addition, deficiency of Bif-1, a membrane curvature promoting protein, can lead to the expansion of adipose mass, reduce the basal rate of adipose tissue lipolysis and induce obesity in mice. Bif-1 deficiency also reduces the abundance of autophagic-lysosomal proteins Atg9 and Lamp1 (Liu et al., 2016).

## Conclusion

The field of lipophagy has yet to fully develop. Despite its infancy, it has already managed to provide significant new insights on lipid metabolism and energy homeostasis and represents a promising path forward to advance our understanding of lipid-associated disorders. Further research on the exact mechanisms of lipophagy regulation is certain to reveal valuable new targets for therapeutic approaches and improve our available toolset against obesity, liver disease and cancer.

## Author Contributions

KK and MC were responsible for generating the primary draft and the graphics. MM and NT contributed to the organization, suggestions on the content and editing of the manuscript.

## Conflict of Interest Statement

The authors declare that the research was conducted in the absence of any commercial or financial relationships that could be construed as a potential conflict of interest.
